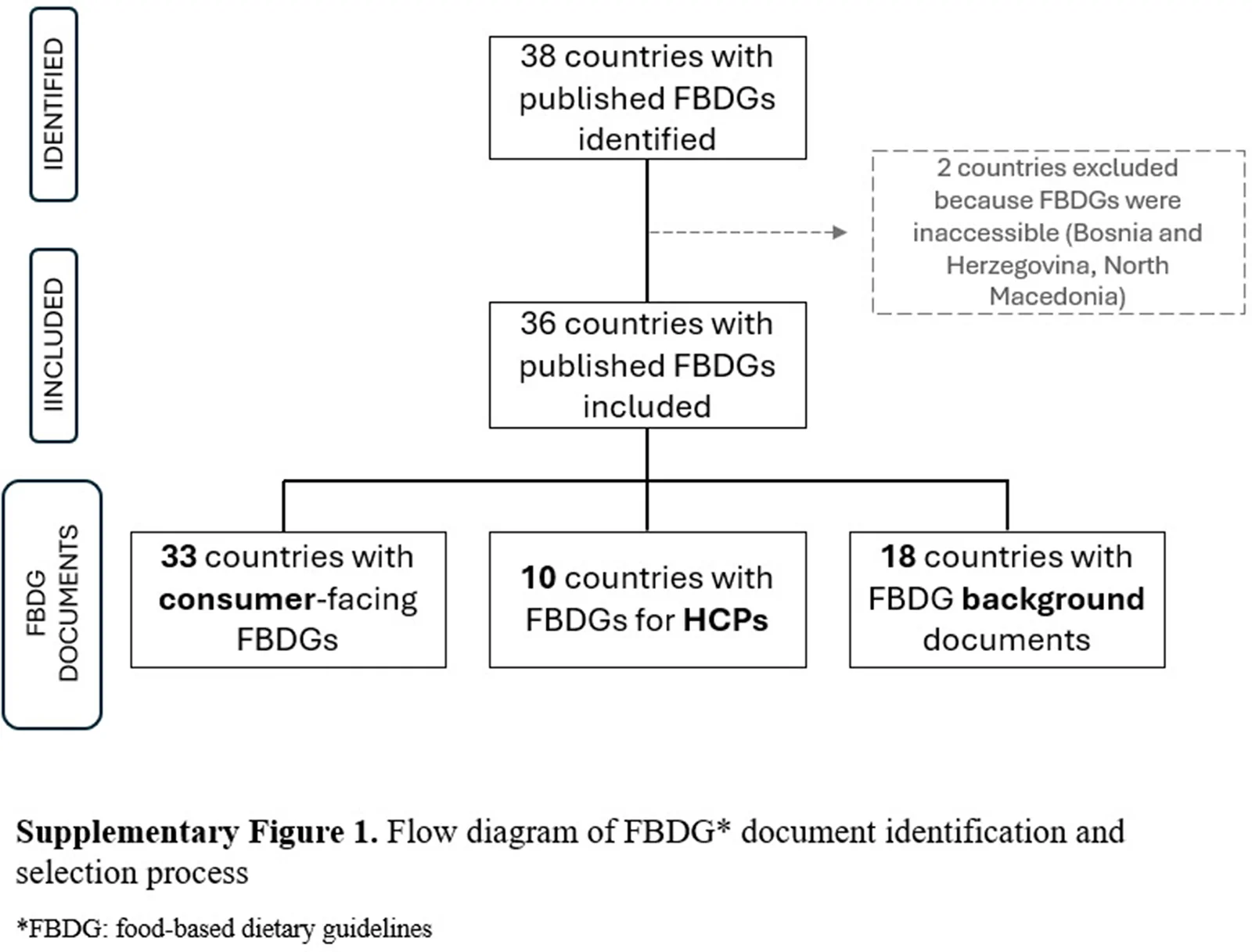# Examination of Sustainability Messaging in European Food-Based Dietary Guidelines

**DOI:** 10.1016/j.cdnut.2026.109440

**Published:** 2026-07-20

**Authors:** Fanny Salesse, Rebecca Finlay, Samara Jones, Alison L Eldridge, Eileen R Gibney

**Affiliations:** 1 UCD Institute of Food and Health, University College Dublin, Belfield, Dublin, Ireland; 2 Insight Research Ireland Centre for Data Analytics, University College Dublin, Belfield, Dublin, Ireland; 3 Co-Centre for Sustainable Food Systems, University College Dublin, Belfield, Dublin, Ireland; 4 Nestlé Institute of Health Sciences, Nestlé Research, Lausanne, Switzerland

**Keywords:** environment, sustainability, food-based dietary guidelines (FBDGs), plant-based diets, public health nutrition

## Abstract

**Background:**

Shifting to more sustainable diets can reduce environmental impact and improve population health. Food-based dietary guidelines (FBDGs) offer an opportunity to incorporate sustainability into nutritional recommendations.

**Objectives:**

This study primarily examined the inclusion of environmental sustainability messaging across European FBDGs. A secondary analysis assessed how such messaging was associated with recommendations for 2 key food groups relevant to the transition toward sustainable healthy diets, namely meats and pulses.

**Methods:**

Three FBDG document types were reviewed: consumer-facing guidelines, resources for health care professionals, and scientific background reports. Eighteen sustainability criteria derived from Food and Agriculture Organization/World Health Organization (FAO/WHO) and the British Dietetic Association’s recommendations were examined. Criteria were coded as explicitly linked to sustainability, included without sustainability reference, or missing. Recommended daily intakes for total, red and processed meat, and pulses were extracted from consumer-facing documents where available.

**Results:**

FBDGs from 36 countries were analyzed. Guidelines referencing sustainability were generally more recently published, with explicit sustainability messaging most commonly found in scientific background documents. Across document types, criteria related to reducing animal-based foods, increasing plant-based foods, and minimizing environmental impact were most frequently included. Guidance related to locally produced or seasonal foods was included in consumer-facing materials, whereas recommendations regarding food waste were rarely mentioned. FBDGs that included messages promoting plant-based foods generally recommended lower meat and higher pulse intakes, although trends between messages and recommended amounts were not always consistent. For example, messages related to reducing animal foods, animal protein, or red meat were not consistently associated with lower recommended intakes of total meat. However, FBDGs specifically addressing red or processed meat intake generally recommended lower red and processed meat consumption and higher pulse intakes.

**Conclusions:**

Overall, European FBDGs increasingly incorporate environmental sustainability, but gaps remain. Consumer-facing guidance remains largely health-focused. Greater coherence is needed in the translation of sustainability principles into FBDGs.

## Introduction

Food systems encompass all elements and activities that relate to the production, processing, distribution, preparation, and consumption of food and the outputs of these activities, including socioeconomic and environmental outcomes [1,2]. Globally, current food systems have a considerable impact on the environment [3,4], causing environmental degradation and accelerated climate change, and often fail to adequately meet the nutritional needs of many regions [5,6].

In recent years, the relationship between diets and environmental sustainability has received increasing attention [6,7]. In 2023, the Stockholm Resilience Centre published an update on planetary boundaries highlighting that 6 of 9 boundaries were exceeded [8], with food systems being the main driver of excess for 5 of them [9]. In parallel, the Global Burden of Disease Study highlighted unhealthy dietary patterns lead to 11 million deaths and 255 million disability-adjusted life years annually [10,11]. These primarily stem from diet-related chronic diseases that also result in higher healthcare costs [12]. Indeed, globalization has contributed to a “nutritional transition” toward Western diets, characterized by high intakes of animal-sourced foods, refined sugar, processed foods and fats, and low intakes of fruits, vegetables, and whole grains [13]. The production of these foods is energy-intensive and relies heavily on nonrenewable inputs, contributing to environmental damage [[14], [15], [16]]. Food production processes account for over a third of global greenhouse gas emissions (GHGEs) [17,18]. As the world population is expected to increase from 8 billion currently to 10 billion around 2080 [19], global food production is anticipated to rise by 15% in the forthcoming decades, which may have an impact on food system GHGEs [20].

A crucial move toward more sustainable food systems is therefore needed. Several measures have been implemented to help develop guidelines and recommendations on what constitutes a healthy and sustainable diet [21]. The UN Sustainable Development Goals include goals for sustainable agriculture, consumption and production patterns, and combating climate change and its impacts [22]. In 2019, the FAO and the WHO jointly held a Consultation on Sustainable and Healthy Diets in Rome [21]. In the same year, The EAT-Lancet Commission report promoted universal nutrition guidelines, called the Planetary Health Diet. This report highlighted the importance of implementing a universal framework that each country can adapt to their national food-based dietary guidelines (FBDGs) [5]. The release of the EAT-Lancet report 2.0 in October 2025 highlighted the pivotal role of updating guidance on healthy and sustainable diets [9].

A key action to ensure healthy and sustainable diets are available, accessible, affordable, safe, and desirable is the creation of national FBDGs based on these principles (FAO/WHO) [23]. To achieve this, FBDGs are an essential public health tool to promote healthy diets and lifestyles for the general population and often form the basis of national nutrition policies. FBDGs include recommendations on foods, food groups, and dietary patterns based on scientific evidence while also taking cultural context into account [24]. Apart from influencing the food environment at a national level, FBDGs carry global repercussions, especially when recommending consumption patterns that may contradict the achievement of international environmental goals [25]. Therefore, they represent an opportunity to support the population in following sustainable and healthy diets [25,26].

As early as 2016, the FAO stated the importance of developing and disseminating FBDGs that stem from both health and sustainability objectives [27]. Shortly after, scientists concluded that future FBDG development should place greater emphasis on the environmental impacts of diets [24,28]. However, recent reviews have shown that most FBDGs continue to fall short of recommending reductions in the consumption of food groups associated with high GHGEs [29], and explicit guidance on environmentally sustainable dietary practices remains largely absent from consumer-facing materials [30].

Although most reviews have focused on consumer-facing FBDG documents, these represent only one stage of a broader evidence translation process in the development of FBDGs [31,32]. National FBDGs are typically developed through several layers of evidence, beginning with scientific or technical reports that synthesize the evidence base, which are then translated into guidance materials for health professionals and the public [31]. Sustainability considerations may therefore appear or disappear or be more explicitly articulated at different stages of this process.

Given the rapid pace at which FBDGs are being revised, it is essential to monitor their evolving content to identify emerging trends and assess progress toward the inclusion of sustainability principles. Building on these observations, the present study first aimed to examine the extent to which environmental sustainability messaging is incorporated into European FBDGs, using a structured set of sustainability criteria. A second objective was to evaluate the association between the presence of these messages in the FBDGs and the impact of these recommendations on recommended daily amounts of consumption for meat and legumes/pulses, considered as pivotal food groups in the shift toward more sustainable and healthy diets [5,33,34]. The analysis focused on FBDGs published in Europe, as this region has the highest number of countries with existing guidelines [35].

## Methods

A systematic approach was used to identify, collect, and analyze FBDGs across countries in the FAO European region. The methods comprised 3 main components: *1*) identifying and reviewing FBDG documents; *2*) assessing the inclusion of sustainability-related messaging within these guidelines based on 2 established frameworks(the Sustainable Healthy Diets Guiding Principles and the One Blue Dot Sustainable Diet Recommendations); and *3*) extracting and analyzing quantitative recommendations for meat and legume/pulse consumption. Only the recommendations aimed toward the general adult population were included in this analysis; FBDG documents aimed at children and/or pregnant/lactating women or older adults were not included.

### FBDG documents

A search for European FBDGs and supporting documents was conducted between 25 July, 2025, and 10 August, 2025. The FAO’s online repository of FBDGs [35] and the European Commission’s Health Promotion and Disease Prevention Knowledge Gateway [36] were used to identify countries with published FBDGs. Where possible, the source documents were obtained from the links provided in these repositories. When required, additional searches to find the relevant FBDG documents were completed. This ensured the most recent documents were used in the data extraction and analysis. A Google search was also conducted to find newer versions of any FBDGs, using the following terms: “food-based dietary guidelines + [country],” “nutrition recommendations + [country],” and “[country] + ministry of health.” To identify background documents, which are sometimes published separately from the FBDGs, an additional search was conducted using: “food-based dietary guidelines + [country] + background document” and “food-based dietary guidelines + [country] + scientific report.”

Three types of FBDG documents were reviewed: consumer-facing documents, health care professional (HCP) documents, and background/scientific reports/documents (Supplemental Table 1). These were defined as follows: *1*) consumer documents were typically the main documents, written in lay language, and often used graphics to depict the recommendations; *2*) the HCP documents offered scientific details supporting the nutritional guidance presented in the consumer documents; and *3*) the background documents provided a description of the methodology used to develop the guidelines, or the scientific rationale. If, for the same country, > 1 FBDG document fell into 1 of the 3 categories, all relevant documents were reviewed, and their data were then combined.

Following identification and access of the documents, Google Translate (Google Translate LLC, 2023) was used to translate documents that were not available in English. A Microsoft Excel spreadsheet (Version 2401) was developed to compile the extracted data using both established sustainable diet criteria, as detailed below. All FBDG documents were systematically screened by 2 independent reviewers to determine if, and how, they addressed each individual criterion listed in the sustainability framework. Each criterion was considered within each document and classified into 1 of 3 categories: *1*) included with a direct reference to environmental sustainability (explicit message); *2*) included without reference to environmental sustainability (general message); or *3*) not mentioned. In cases of uncertainty regarding whether an FBDG met a given criterion, discussions were held between the research team (RF and FS) to reach consensus. Additional information extracted included the year of publication, whether sustainability was mentioned, and whether a related term was used, based on the following search terms: “sustainability,” “sustainable,” “environment∗,” “climate,” and “planet∗.”

### Sustainability criteria

An initial search was conducted to find existing guidelines on healthy and sustainable diets using the search engines PubMed, Google Scholar, and OneSearch. The search yielded a number of guidelines, 2 of which were selected to form the basis of the sustainability inclusion criteria used in this review. The first set of selected guidelines was the Sustainable Healthy Diets Guiding Principles developed by the FAO and the WHO [22]. This set of 17 goals was selected as it provides universally applicable guidelines to achieve sustainable diets and address malnutrition, serving as a framework for policymakers worldwide. It sets out 16 principles of healthy and sustainable diets belonging to 3 categories, namely health, environmental impacts, and sociocultural aspects. For the purpose of this study, these were amended to exclude the first guiding principle about breastfeeding being preferred because the focus was on adults, and the sociocultural criteria were excluded as they were not within the scope of this study. The second set was The One Blue Dot Sustainable Diet Recommendations from the British Dietetic Association (BDA) [37]. These were developed as part of the One Blue Dot project based on the Sustainable Diets Policy, which aims to translate national and international guidelines on healthy and sustainable diets. It was selected because it is similar to the FAO’s Guiding Principles while demonstrating applicability to the European region. In addition, the BDA’s guidelines were initially created for dietitians and therefore provide more diet- and food-specific sustainable recommendations.

Across these 2 sets of guidelines, a total of 18 sustainability criteria were considered, including recommendations to choose local or seasonal fruits and vegetables, increase consumption of whole grains, reduce consumption of red and processed meats, limit consumption of processed foods and beverages high in fat, salt, and sugar (HFSS), moderate dairy consumption and consume dairy alternatives, recycle waste, and minimize food packaging. The 18 adapted criteria included in this study were mapped to the original terminology used in the source documents (FAO or BDA), as shown in Supplemental Table 2.

For each country, all relevant FBDG documents were reviewed against these criteria and categorized as described previously. The number of mentions was tabulated across 38 countries and assessed separately for consumer documents, documents for HCPs, and scientific background documents. All criteria were weighted equally.

### Total meat, red and processed meat, and pulse recommendations

To identify the association between the presence of environmental sustainability messaging and recommendations for meats and legumes/pulses, total recommended daily gram amounts of meat (including poultry), red and processed meat, and legumes/pulses were extracted from the consumer-facing FBDG documents, where available. These food groups were selected because they are broadly representative of environmental considerations to reduce meat (in particular, red and processed meats) and increase plant-based food consumption consistent with the EAT-Lancet recommendations. Meat, particularly red and processed meat, is widely recognized as a major contributor to GHGEs, whereas pulses are commonly promoted as a sustainable, nutrient-dense, and cost-effective plant protein alternative [38,39]. Other plant-based protein sources, such as nuts, seeds, and wholegrain cereal-based products, were less consistently represented as such across FBDGs and were therefore not included in this secondary analysis.

Values were obtained either directly from stated daily totals or indirectly derived from weekly totals and/or from guidance combining portion size and recommended consumption frequency. Because many FBDG documents do not clearly specify whether recommended meat quantities refer to raw or cooked weights, it was assumed that reporting may vary across guidelines. For meat, where both raw and cooked values were available, they were averaged to ensure harmonized reporting and comparability across FBDGs, whereas for pulses, cooked weights were retained when provided. Therefore, reported amounts for legumes and pulses refer to cooked weights, whereas meat values represent the mean of raw and cooked weights when both were provided.

The detailed methodology used to derive portion sizes and frequencies has been described previously [40].

## Results

### Identified FBDG documents

**FIGURE 1:**
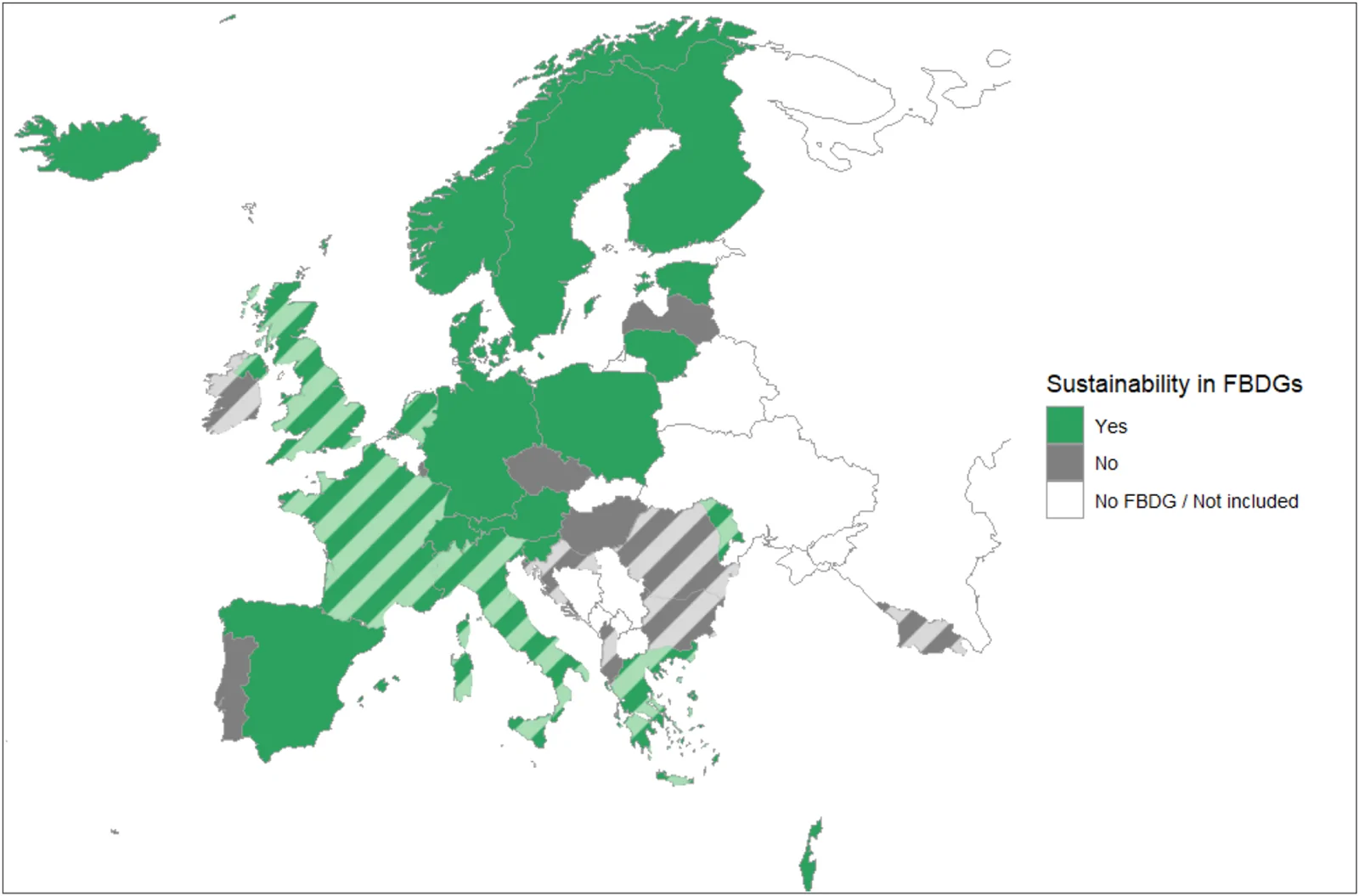
Map of European consumer-facing food-based dietary guidelines (FBDGs) with mention of environmental sustainability or a similar term (*n* = 33). Stripes indicate a publication date before 2020.

A total of 38 FBDGs from European countries were identified (Supplemental Figure 1). Of these, 2 were excluded because the source documents were not accessible (Bosnia and Herzegovina and North Macedonia). Of the remaining 36, 33 included consumer-facing FBDGs, 10 included documents for HCPs, and 18 countries included more extensive scientific evaluation source documents. Of these, 5 countries included all 3 types of FBDG documents, 2 included consumer and HCP documents, and 13 included consumer and background documents. The geographic distribution of countries with consumer-facing FBDGs mentioning “sustainability” or a related term is shown in Figure 1. The mean year of publication of consumer-facing FBDGs mentioning sustainability or a related concept was 2021, whereas that of FBDGs not referring to sustainability was 2015. Among consumer-facing FBDG documents, references to sustainability or related concepts were more common in those published since 2020 (*n* = 19) than in earlier documents (*n* = 14). Specifically, 14 of the 19 FBDGs published since 2020 (73.7%) referred to environmental sustainability or a similar concept, compared with 6 of the 14 FBDGs (42.8%) released before 2020.

### Presence of sustainability criteria in FBDG documents

Across all document types, the messages “Minimize environmental impact and preserve biodiversity” (direct mention: 46% in consumer, 70% in HCP, 89% in background documents) and “Reduce consumption of animal-based foods/animal protein and increase plant protein/plant-based foods” (46%, 50%, and 72%, respectively) were the most frequently included with reference to environmental sustainability (Table 1). In both consumer-facing and HCP documents, “Fruits and vegetables: locally produced” (42% and 40%, respectively) was also among the most frequently mentioned messages with regard to sustainability, along with “Fruits and vegetables: choose from seasonal sources” in consumer-facing documents (42%). As seen in Table 1, consumer and HCP materials typically included fewer explicit sustainability-related messages (4.97 and 3.70 criteria, respectively), compared with scientific background documents (9.06 criteria with explicit mention of sustainability, on average).

**TABLE 1:** Number and percentage of mentions for sustainability-related criteria

Criteria	Consumer documents (*n* = 33)			HCP documents (*n* = 10)			Scientific background documents (*n* = 18)		
	Explicit message	General message	Not mentioned	Explicit message	General message	Not mentioned	Explicit message	General message	Not mentioned
Diverse food consumption	8 (24.2)	23 (69.7)	2 (6.1)	1 (10.0)	8 (80.0)	1 (10.0)	8 (44.4)	5 (27.8)	5 (27.8)
Reduce processed meat consumption	13 (39.4)	15 (45.5)	5 (15.2)	1 (10.0)	8 (80.0)	1 (10.0)	9 (50.0)	7 (38.9)	2 (11.1)
Reduce red meat consumption	11 (33.3)	14 (42.4)	8 (24.2)	2 (20.0)	6 (60.0)	2 (20.0)	12 (66.7)	4 (22.2)	2 (11.1)
Limit processed and HFSS foods	8 (24.2)	24 (72.7)	1 (3.0)	2 (20.0)	8 (80.0)	0 (0.0)	12 (66.7)	5 (27.8)	1 (5.6)
Hydration: water as fluid of choice, choose water over soft drinks	10 (30.3)	21 (63.6)	2 (6.1)	1 (10.0)	8 (80.0)	1 (10.0)	10 (55.6)	5 (27.8)	3 (16.7)
Dairy: moderate consumption	4 (12.1)	4 (12.1)	25 (75.8)	2 (20.0)	2 (20.0)	6 (60.0)	11 (61.1)	1 (5.6)	6 (33.3)
Consume dairy alternatives	4 (12.1)	2 (6.1)	27 (81.8)	1 (10.0)	3 (30.0)	6 (60.0)	2 (11.1)	6 (33.3)	10 (55.6)
Increase wholegrains	12 (36.4)	20 (60.6)	1 (3.0)	2 (20.0)	8 (80.0)	0 (0.0)	10 (55.6)	6 (33.3)	2 (11.1)
Reduce food loss and waste	11 (33.3)	3 (9.1)	19 (57.6)	2 (20.0)	2 (20.0)	6 (60.0)	13 (72.2)	0 (0.0)	5 (27.8)
Reduce consumption of animal-based foods, reduce animal protein, increase plant protein, increase plant-based foods	15 (45.5)	14 (42.4)	4 (12.1)	5 (50.0)	3 (30.0)	2 (20.0)	13 (72.2)	3 (16.7)	2 (11.1)
Prefer sustainably sourced fish	13 (39.4)	1 (3.0)	19 (57.6)	3 (30.0)	0 (0.0)	7 (70.0)	11 (61.1)	0 (0.0)	7 (38.9)
Adequate nutrition, limit overconsumption	3 (9.1)	16 (48.5)	14 (42.4)	1 (10.0)	5 (50.0)	4 (40.0)	6 (33.3)	5 (27.8)	7 (38.9)
Animal production: minimize antibiotics and hormones	0 (0.0)	0 (0.0)	33 (100.0)	0 (0.0)	0 (0.0)	10 (100.0)	2 (11.1)	0 (0.0)	16 (88.9)
Minimize food packaging	6 (18.2)	1 (3.0)	26 (78.8)	0 (0.0)	0 (0.0)	10 (100.0)	6 (33.3)	0 (0.0)	12 (66.7)
Minimize environmental impact, biodiversity preservation	15 (45.5)	0 (0.0)	18 (54.5)	7 (70.0)	0 (0.0)	3 (30.0)	16 (88.9)	0 (0.0)	2 (11.1)
Fruits and vegetables: choose from seasonal	14 (42.4)	9 (27.3)	10 (30.3)	3 (30.0)	3 (30.0)	4 (40.0)	8 (44.4)	2 (11.1)	8 (44.4)
Fruits and vegetables: locally produced	14 (42.4)	7 (21.2)	12 (36.4)	4 (40.0)	2 (20.0)	4 (40.0)	11 (61.1)	1 (5.6)	6 (33.3)
Recycle food waste	3 (9.1)	0 (0.0)	30 (90.9)	0 (0.0)	0 (0.0)	10 (100.0)	3 (16.7)	0 (0.0)	15 (83.3)

Abbreviations: HCP, health care professional; HFSS, high in fat, salt, and sugar.

Values are expressed as n (%).

In comparison to messages related to protein sources or wholegrain intake, consumer-facing FBDGs rarely addressed dairy consumption with respect to sustainability (i.e., recommendations to moderate dairy intake or to consume dairy alternatives were only present in 24.2% and 18.2% of documents, respectively). Supplemental Table 3 provides these detailed numbers for each criterion from the 2 sets, separately (FAO and BDA).

**FIGURE 2:**
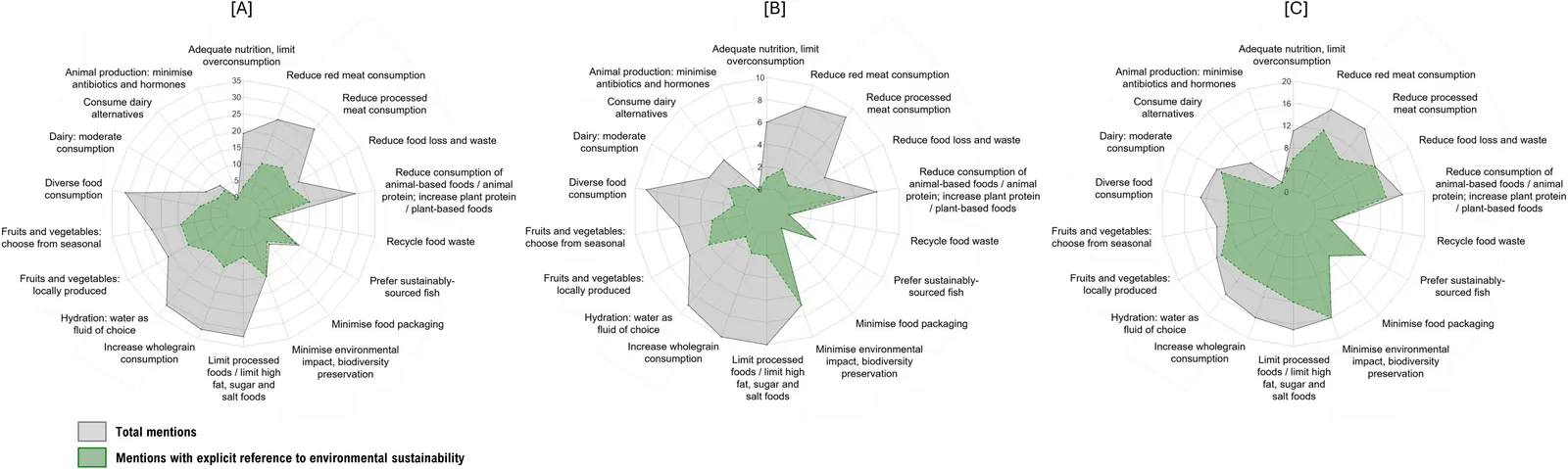
Total number of mentions for each criterion (grey area), of which explicit sustainability mentions are shown in green. (A) Consumer documents (*n* = 33). (B) Health care professional documents (*n* = 10). (C) Background documents (*n* = 18).

Figure 2 presents the total number of mentions for each message, distinguishing those with explicit mention of environmental sustainability in green and those with general environmental sustainability messaging in grey. This figure illustrates that a higher proportion of criteria were included with direct reference to sustainability in background documents compared with consumer and HCP documents across all 18 criteria. Overall, across the 18 criteria, explicit mentions of environmental sustainability were found in 28% of consumers FBDGs, 21% of HCP FBDGs, and 51% of background documents.

The criteria mentioned least frequently across all document types were “Recycle food waste” (explicit mention of environmental sustainability: 9% in consumer, 0% in HCP, 17% in background) and “Animal production: minimize antibiotics and hormones” (0%, 0%, and 11%, respectively). In contrast, consumer and HCP documents more often included general criteria without reference to sustainability, particularly “Diverse food consumption” (general mention: 70% in consumer, 80% in HCP documents) and “Limit processed foods and HFSS foods” (73% and 80%, respectively).

### FBDG recommendations for pulses and meat and their alignment with sustainability criteria

**FIGURE 3:**
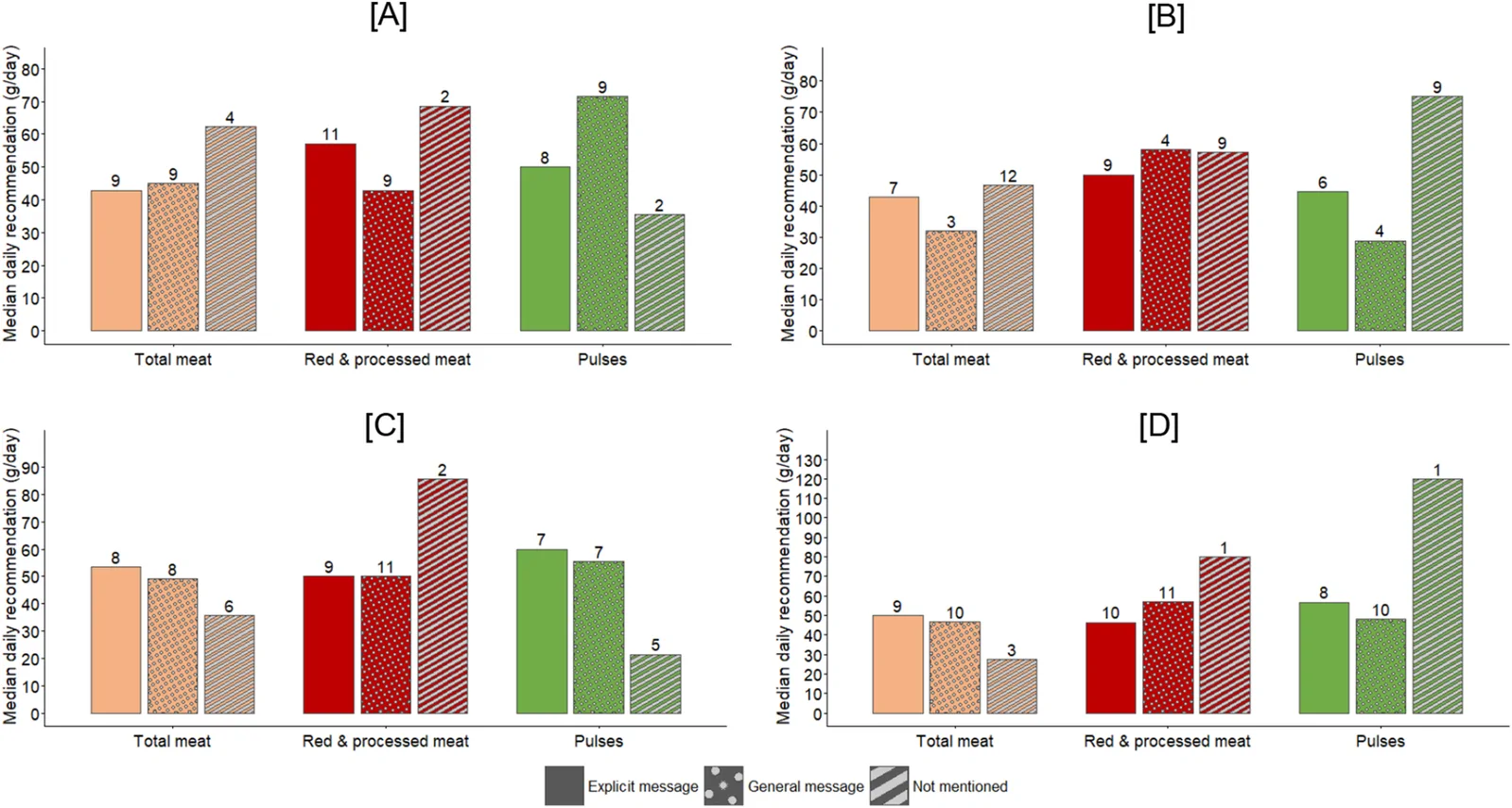
Recommended amounts per day for total meat, red and processed meat, and pulses, and sustainability criteria inclusion in consumer-facing food-based dietary guidelines. Numbers above columns indicate sample size. (A) Increase plant-based foods/protein. (B) Reduce animal foods/protein. (C) Reduce red meat. (D) Reduce processed meat.

Notable differences emerged in the median recommended daily amounts of meat and pulses depending on the type of guidance provided. Figure 3 presents the recommended daily intakes for total meat, red and processed meat, and pulses according to the inclusion of various criteria. In FBDGs using messages related to increasing either plant-based protein or plant-based foods (Figure 3A), the recommended total meat intake was substantially higher when the criterion was not mentioned (62.3 g/d) compared with guidelines using a general message (45.0 g/d) or those explicitly referring to environmental sustainability (42.9 g/d). In contrast, recommended intakes for pulses were higher when either criterion (increase plant-based protein or increase plant-based foods) was mentioned (50 g/day) than when they were not (35.5 g/d) and were highest in guidelines including a general message (71.4 g/d).

For FBDGs including “reducing animal foods/animal protein,” the recommended amounts for total meat and for red and processed meat varied little, although recommendations for pulses were higher in guidelines in which the criterion was not mentioned (Figure 3B).

Across documents that included messages targeting a limitation or reduction of red meat intake, recommendations showed higher total meat intakes, lower red meat intakes, and higher pulse intakes compared with guidelines that did not include such messages (Figure 3C). Finally, in FBDGs recommending limiting or reducing processed meat intake (Figure 3D), the recommended daily intake of total meat was higher, while the recommendations for red and processed meat, as well as for pulses, were lower compared with guidelines that did not mention this criterion.

## Discussion

This study assessed the presence of environmental sustainability messages in FBDGs from European countries and examined whether the inclusion of such messages was associated with adjusted recommended daily amounts of meat and pulses. We found that more recent FBDGs were more likely to align with the guiding principles and explicitly refer to environmental sustainability, suggesting that ecological considerations are increasingly being incorporated into national dietary guidance. However, the extent and nature of this integration varied considerably across document types, being notably less represented in consumer-facing and HCP materials.

Not all sustainability criteria identified by the FAO [22] and BDA [37] were reflected in European FBDGs. Some criteria with a clear environmental focus (e.g., preserve biodiversity, choose sustainably sourced fish) were rarely mentioned, whereas broader, predominantly nutrition-oriented criteria (e.g., limit processed foods, diverse food consumption) appeared much more frequently. These latter recommendations appear to be generally framed around health outcomes, with environmental benefits remaining implicit.

By examining not only consumer-facing FBDGs but also HCP-focused and scientific background documents, this research offered a more complete picture of how sustainability is embedded throughout the guideline development and communication pathway. Background scientific documents typically included more explicit and detailed references to environmental sustainability than either HCP or consumer-facing documents, reflecting their role as technical foundation documents rather than public communication tools. As HCP materials act as intermediaries between policy and the population, communication directed at the public remains primarily health-oriented, even when policy foundations are sustainability-driven. Only 5 of the 19 consumer materials published since 2020 referred to the term “sustainability” or a synonym. The mention of sustainability—or lack thereof—may reflect government priorities or preferred communication approaches. This framing shows that messages tied to health outcomes may be more tangible and resonate more strongly with the public than those framed primarily around planetary health [41]. In a recent review of factors influencing sustainable and healthy diet consumption, the authors used an individual scoring system in which “health consciousness” ranked as a higher priority for intervention than “concern for environment” (note: healthy eating intentions ranked at the same level as the latter) [42]. Other studies have shown that using health-oriented messages to communicate about environmental risks increased public engagement on these topics and support for climate policies [43,44].

We also observed variation in the accessibility of sustainability-related messages. In some FBDGs, environmental aspects were confined to a specific section, a separate document, or a distinct “sustainability” tab, rather than being part of the main core dietary advice. For example, the Danish guidelines, titled “The Official Dietary Guidelines - good for health and climate,” emphasized the integration of environmental sustainability throughout the document [45], whereas the Austrian messages around sustainability were restrained to a separate web page of the guidelines [46]. Although these messages were technically present, they may be difficult to locate for certain users. Therefore, the prevalence of sustainability messages discussed in this paper may not fully reflect their emphasis or prominence within the guidelines. In fact, it is important to note that FBDG recommendations are the result of elaborate processes, sometimes considering competing scientific, social, and political factors [31].

Our findings also support the notion that many sustainability-related criteria overlap with messages that commonly promote health. Recommendations such as limiting processed or HFSS foods, encouraging diverse dietary patterns or increasing fruit, vegetable, and wholegrain intake are well established in health-focused guidelines and are largely congruent with sustainable diet patterns [47,48]. In our coding, many of these criteria were mentioned without any explicit environmental framing, even when they aligned strongly with sustainability goals. This overlap raises both opportunities and challenges. On one hand, it is possible that promoting dietary patterns that are simultaneously “healthy” and “sustainable” may increase clarity and reduce message fatigue. In addition, consumers’ motivations toward health and environmental benefits tend to converge, as sustainable foods are often perceived as healthier and more nutritious and vice versa [[49], [50], [51]]. Therefore, the promotion of more sustainable food choices could also resonate with consumers in terms of health benefits. On the other hand, the lack of explicit environmental considerations may limit public understanding that dietary choices have environmental consequences, with such a lack of awareness being an important barrier to the adoption of sustainable diets [52].

Several food groups are worth discussing here with respect to sustainability. By exploring associations between the inclusion of specific sustainability-related criteria and recommended gram-per-day amounts for meat and pulses, we provide preliminary insights into whether sustainability discourse is reflected in the quantitative aspects of dietary guidance. The results suggest that the inclusion of sustainability criteria may substantially influence the quantitative dietary advice provided in FBDGs, but not always in predictable or coherent ways. This raises questions about consistency and evidence translation in the guideline development process. For meat and red and processed meat, the deeply anchored meat culture in some countries may necessitate more targeted approaches that emphasize that intake can be reduced without requiring complete elimination of the food from the diet [53]. With regard to plant-based foods, the placement and treatment of pulses within the depicted “food plate” may further influence both recommendations and their interpretation. In a global review of the inclusion of plant-based diets and substitutions for animal-based foods in FBDGs, Klapp et al. [54] highlighted that about half of countries classified legumes separately from the protein-rich food group (which generally comprises meat, fish and eggs). According to the authors, a reclassification with the creation of more “inclusive” food groups could make pulses more visible as a protein alternative to meat and may therefore better support shifts toward plant-based diets [54]. In our study, the sample of the recommended amounts was small; therefore, conclusions remain limited. Further work using a larger set of guidelines (e.g., including other global regions) is warranted to study the optimal approaches for the provision of qualitative and/or quantitative guidance in FBDGs. Finally, few FBDGs promoted moderation of dairy consumption, and those that did generally justified this recommendation by the fat content of cheese. Plant-based dairy alternatives were infrequently mentioned and, if so, often accompanied by caveats (e.g., the need to opt for fortified products). This finding aligns with previous work indicating that FBDG developers may hesitate to recommend reduced dairy intake due to “complex nutritional implications” and concerns about micronutrient adequacy at a population level [29]. However, this is unlikely to be unique to dairy, as sustainability considerations may also be influenced across other food groups (such as meat) by broader economic, political, and cultural factors discussed within working groups as part of FBDG development. Stakeholder input from HCPs, consumer groups, and industry representatives may therefore influence governmental priorities [30].

Overall, our results are broadly consistent with previous studies examining the integration of sustainability into FBDGs, which have reported limited but growing inclusion of environmental considerations and substantial heterogeneity across countries [23,25,30]. Compared with more global frameworks (e.g., EAT-Lancet), most European FBDGs tend to remain conservative in their recommendations regarding reductions in animal-sourced foods and increases in plant-based foods. This suggests that other aspects of sustainability, not related to environmental sustainability, may also be prioritized. For example, affordability, social acceptability, and justice were emphasized in EAT-Lancet 2.0 [9]. Policy makers may therefore prefer to provide practical tips on saving money when shopping or on meal planning, rather than explicitly advocating for sustainable consumption. Although this analysis focused on European FBDGs, developments in other regions reinforce the broader trend toward integrating sustainability into dietary guidance. The authors of a recent review concluded that European and Central Asian countries were more likely to reference environmental sustainability in their guidelines than countries in South Asia, East Asia, and the Pacific regions. The focus on environmental sustainability was generally found to be predominant in upper-middle–income and high-income countries (34 of 72), while at that time, only 27% of the guidelines in low-income or lower-middle-income countries addressed sustainability [30].

A key strength of this study is the systematic and harmonized approach used to identify and code sustainability criteria across multiple document types and countries. However, several limitations should be acknowledged. First, despite efforts to standardize coding decisions between reviewers, interpretation of complex policy documents inevitably involves a degree of subjectivity. Additionally, the possibility of minor inaccuracies pertaining to the translation of certain documents from their original language to English via Google Translate cannot be excluded. In this work, all documents were examined in duplicate, and when conflicts arose, consensus was achieved by discussion with a third author. Despite this, the selection and operationalization of criteria (e.g., what constitutes an explicit compared with a general message) may reflect author judgment. This is especially relevant for scientific background documents, which provide underlying rationale rather than succinct guidance messages and can thus be interpreted in multiple ways. To our knowledge, it is the first study to combine elements from 2 established sets of sustainability criteria (FAO and BDA), allowing a more comprehensive assessment across multiple aspects of environmental sustainability. To avoid overlap, the decision was made to merge some closely related criteria during analysis, for example, “limit HFSS foods” and “limit processed foods,” because of how jointly framed they were in many FBDGs (i.e., messages encouraging a reduction of processed food consumption were often accompanied by examples of HFSS packaged foods). Although this decision improved consistency in coding, it may suppress important distinctions, particularly given that not all ultra-processed foods (UPFs) are HFSS [[55], [56], [57]]. In fact, various alternatives to animal-sourced foods, including plant-based meat alternatives or fortified plant-based milks, fall under the UPF definitions [58,59]. To avoid consumer confusion, messages could focus on recommendations to increase whole-food plant-based food categories rather than a reduction of HFSS foods or UPFs (for example, when recommending to lower consumption of processed meat).

Another limitation to our analysis was the restriction to European FBDGs, which may fail to capture global variability in how sustainability principles are incorporated into dietary guidance. Other regions, such as Africa and Latin America, have recently published dietary guidelines that integrate environmental sustainability [60,61]. Future research should extend this work to a broader set of countries and examine regional similarities and differences.

This study makes several contributions to the emerging literature on sustainability in FBDGs. It provides an updated overview of the integration of environmental sustainability criteria in European guidelines, capturing a recent wave of FBDG updates occurring in the context of heightened public and political attention to climate and environmental issues. With around one-third of the studied consumer-facing documents published between 2024 and August 2025 and additional revisions currently underway, the presence and prominence of sustainability messages are likely to evolve further in the near future. As countries with diverse population profiles revise their FBDGs or develop them for the first time, the incorporation of environmental sustainability, as well as other aspects of sustainability (social, economic), will require additional attention.

## Author contributions

The authors’ responsibilities were as follows – FS, SJ, ERG: design; FS: methodology and formal analysis; FS, RF, SJ, investigation; RF: validation; FS, RF, data curation; FS, RF, SJ: writing – original draft; FS, ALE, ERG: writing – review and editing; ALE, ERG: supervision and funding acquisition; ERG: project administration and responsibility for final content; and all authors: read and approved the final manuscript.

## Data availability

Data described in the manuscript, code book, and analytic code will be made available upon request pending [e.g., application and approval, payment, other].

## Declaration of Generative AI and AI-assisted technologies in the writing process

During the preparation of this work, the authors used ChatGPT, version GPT-4o in order to correct spelling and grammar. After using this tool, the authors reviewed and edited the content as needed and take full responsibility for the content of the publication.

## Funding

The authors declare that financial support was received for the research, authorship, and/or publication of this article. This publication had emanated from research conducted with the financial support of Insight Research Ireland Centre for Data Analytics, under Grant number [12/RC/2289_P2] and the Co-Centre for Sustainable Food Systems co-funded by Research Ireland, Northern Ireland’s Department of Agriculture, Environment and Rural Affairs (DAERA), UK Research and Innovation (UKRI) via the International Science Partnerships Fund (ISPF) under Grant number 22/CC/11147. Ph.D. support for FS was provided by Société des Produits Nestlé, Switzerland.

## Conflict of interest

FS’s PhD is supported by Nestle Research, Societe des Produits Nestle, Lausanne, Switzerland. ALE was an employee of Nestle Research (retired). ERG has received research funding through the following: Food for Health Ireland (www.fhi.ie) project, funded by Enterprise Ireland, co-funded with core partners Carbery, Kerry, Tirlan, Dairygold & Bord Bia. Science Foundation Ireland Co-Centre for Sustainable Food Systems & Insight Centre for Data Analytics; Horizon Europe most recently in projects such as FNSCloud, PLANEAT and MarieCurie CareerFIT, Ph.D. studentship funding from Société des Produits Nestlé, Switzerland. ERG had completed consultancy work for the following: Société des Produits Nestlé, Switzerland; Irish Advertising Standards Agency, Food Safety Authority of Ireland. No personal payment was received; all payments were made into a research fund through Consult UCD. All other authors report no conflicts of interest.

## Figures and Tables

**Figure:**